# The Relationships of Parental Responsiveness, Teaching Responsiveness, and Creativity: The Mediating Role of Creative Self-Efficacy

**DOI:** 10.3389/fpsyg.2021.748321

**Published:** 2022-02-04

**Authors:** Yanna Zhang, Pin Li, Zhitian Skylor Zhang, Xingli Zhang, Jiannong Shi

**Affiliations:** ^1^Research Center of Jiangsu Rural Education Development, Yancheng Teachers University, Yancheng, China; ^2^CAS Key Laboratory of Behavioral Science, Institute of Psychology, Chinese Academy of Sciences, Beijing, China; ^3^Department of Psychology, University of Chinese Academy of Sciences, Beijing, China; ^4^School of Education and Psychology, Chengdu Normal University, Chengdu, China; ^5^Department of Educational Psychology and Research on Excellence, Friedrich-Alexander University, Nuremberg, Germany; ^6^Department of Learning and Philosophy, Aalborg University, Aalborg, Denmark

**Keywords:** environmental responsiveness, parental responsiveness, teaching responsiveness, creative self-efficacy, creativity

## Abstract

This study investigated the relationships between parental responsiveness, teaching responsiveness, and creativity, as well as the mechanism underlying these associations. We collected data from 584 Chinese college students via convenience sampling method and used self-report scales to measure their perceived parental responsiveness, teaching responsiveness, creative self-efficacy, and creativity. We employed structural equation modeling (SEM) to examine the relationships among these variables and the mediation effect. The results revealed that both parental responsiveness and teaching responsiveness were positively related to student creativity. Moreover, creative self-efficacy mediated the relationships of parental responsiveness, teaching responsiveness, and creativity. The findings highlight the significance of responsiveness from parents and teachers on student creativity and verify the potential mediating role of creative self-efficacy. These findings suggest that teachers and parents can foster creativity by providing warm and supportive responses to students’ creative needs.

## Introduction

Creativity is a key concept in human innovation that improves individual competitiveness and drives civilization forward (Hennessey and Amabile, 2010). Scholars have recognized that while creative geniuses have made remarkable and lasting contributions, ordinary people also offer incremental (by comparison) but still essential contributions (Kaufman and Beghetto, 2009).

Researchers have studied creativity for many decades but have reached no consensus on a definition of the term, in part due to its complex nature and diverse expression from different perspectives across multiple disciplines. Many investigations of creativity take one of two directions. One predominant perspective focuses on the premise of a socially valuable product, referring to eminent creativity (also called “Big-C”). Sawyer (2012) defined creativity as “the generation of a product that is judged to be novel and also to be appropriate, useful, or valuable by a suitably knowledgeable social group” (p. 8). This approach posits that only a few people possess creativity, arguing that only “clear-cut, eminent creative contributions” qualify for the classification (Kaufman and Beghetto, 2009), such as the work of Einstein, Beethoven, and other well-known creators. The other predominant perspective focuses on everyday creativity (also called “little-c”), referring to “human originality at work and leisure across the diverse activities of everyday life” (Richards, 2010, p. 190). This category of creativity underscores the vital role of creativity in daily activities among the general population. It implies that creativity can be identified and nurtured in usual settings, such as schools, homes, workplaces, and social venues. Predominantly, everyday creativity can be related to the greater population—laypeople—compared to eminent creators. Moreover, as Richards (2010) argued, “Everyday creativity can be seen as the ground from which (a later and) more publicly celebrated accomplishment can grow” (p. 193). In the present study, we located our interests in everyday creativity and explored the possible daily environment associated with this capability.

In the ecological systems model of creativity development, Yeh (2004) introduced four ecological systems influencing the development of creativity: the microsystem (personal characteristics), the mesosystem (the family and school experiences), the exosystem (organizational environment), and the macrosystem (social milieu). The author contended that creativity is the result of the interaction between individuality (including knowledge, dispositions, skills, and strategies) and environment (including family, school, organization, and social milieu).

Concerning the effect of the environment on creativity, Torrance (1966) noted that the environment’s response to an individual’s curiosity and needs has a significant impact on the development and function of creative capabilities. This notion is consistent with the concept of environmental responsiveness, which emphasizes a sensitive and warm response from the environment to an individual’s needs. In terms of student creativity, the family subsystem and school experience subsystem (i.e., the mesosystem) are the main immediate ecological systems in which they are living in. These two subsystems interact with each other and greatly impact creativity (Yeh, 2004). Due to the significant roles of parents and teachers in family subsystem and school experience subsystem respectively, their supports toward students have a critical effect on students’ creativity. In other words, parents’ and teachers’ responsiveness has a great potential to contribute to a person’s creativity. Notably, environmental responsiveness includes, in addition to parental and teaching responsiveness, responsiveness from other environments such as organizations or the social milieu. In the current study, the term “environmental responsiveness” specifically refers to parental responsiveness and teaching responsiveness.

Parental responsiveness, one of the dimensions of parenting style (Maccoby and Martin, 1983), refers to “the extent to which parents intentionally foster individuality, self-regulation, and self-assertion by being attuned, supportive, and acquiescent to children’s special needs and demands” (Baumrind, 1991, p. 66). This type of responsiveness emphasizes that parents attend to and respond to children’s needs. Specifically, the parental responsiveness includes behaviors such as sensitivity, acceptance, approval, affection, comfort, and involvement (Bogenschneider and Pallock, 2008; Campbell et al., 2017). A large body of literature shows that parental responsiveness is positively related to creativity. For example, Lim and Smith (2008) found that parenting styles characterized by higher levels of acceptance were related to high levels of creativity. Other researchers have found that parenting styles with high responsiveness (i.e., both permissive and authoritative parenting styles) were positively correlated with creativity (Miller et al., 2012; Mehrinejad et al., 2015), whereas a parenting style with low responsiveness (i.e., authoritarian parenting style) was negatively correlated with creativity (Miller et al., 2012; Fearon et al., 2013). These results indicate that parental responsiveness, a key dimension of parenting style, may benefit creativity. Moreover, researchers reported that parental involvement support (Liang et al., 2021) and parental warmth (Guo et al., 2021), which were seen as vital characteristics of parental responsiveness, were positively correlated with creativity. From these findings, parental responsiveness conceivably offers a democratic climate that encourages children to be independent and autonomous in exploring new ideas and situations, potentially providing a basic environment to improve creativity.

Several investigations have applied Baumrind’s typologies of parenting styles to teaching styles (Walker, 2008; Baker et al., 2009). Teaching responsiveness, one dimension of teaching style, refers to being sensitive and responsive to students’ needs and exhibiting warmth toward students (Dever and Karabenick, 2011; Ertesvag, 2011). Teaching responsiveness also benefits students’ creativity. According to Amabile (1989), teachers’ behaviors can benefit creativity by making students feel worthy, loved, and respected; serving as resources and instructors; and making students feel that they can openly discuss their problems. In the result of a literature review, Davies et al. (2013) concluded that teachers’ awareness of students’ needs and respectful teacher–student relationships support the development of creativity in students. Richardson and Mishra (2018) added that a teacher-created learning atmosphere that is respectful, caring, and tolerant of differences contributes to student creativity. According to these findings, a respectful, caring, and supportive classroom climate established by teachers is significant for student creativity. Such a climate corresponds to the nature of teaching responsiveness. Furthermore, other researchers directly found that perceived teacher support identified as giving students the opportunity, resources, and encouragement to explore the unknown, was positively associated with creativity (Du et al., 2019; Gao et al., 2020). Thus, in the present study, we hypothesize that teaching responsiveness is positively related to creativity.

The literature discussed to this point suggests that parental responsiveness and teaching responsiveness both contribute to creativity. However, how these types of responsiveness facilitate creativity has yet to be investigated. The ecological systems model of creativity development implies that the family subsystem and the school experience subsystem influence creativity through personal characteristics (Yeh, 2004). This model brings new insight into the psychological mechanism of the effect of environmental responsiveness on creativity. In the work domain, in terms of personal characteristics, creative self-efficacy is often tested as an essential mediating variable through which environmental and personal factors build employee creativity (Liu et al., 2016). Similarly, in the area of student creativity, creative self-efficacy is also regarded as a mediating variable in investigating the mechanism between teacher support and creativity (Sun et al., 2021) or the mechanism between supervisory style and creativity (Gu et al., 2017). Both social cognitive theory (Bandura, 1997) and the empirical literature (Rosenfeld et al., 2000) indicate that parents and teachers are crucial sources of students’ self-efficacy. Additionally, creative self-efficacy is correlated with creativity (Li and Wu, 2011; Puente-Diaz and Cavazos-Arroyo, 2017; Royston and Reiter-Palmon, 2019). These studies suggest that creative self-efficacy may be a process variable and mediate the associations between parental responsiveness, teaching responsiveness, and creativity.

Creative self-efficacy originates from the general definition of self-efficacy in terms of the targeted perceived ability (Bandura, 1997). This term is defined as a person’s belief in their capability to produce creative outcomes (Tierney and Farmer, 2002). When engaging in creative activities, individuals often face discouragingly slow progress, highly uncertain outcomes, and socially devalued creations, requiring unshakable confidence to persevere in creative endeavors (Bandura, 1997). According to social cognitive theory, strong creative self-efficacy tends to enhance individuals’ perseverance, allowing them to achieve creative outcomes, a proposition first supported by Tierney and Farmer (2002). Extensive studies set in many domains later demonstrated similar positive relationships (Li and Wu, 2011; Jaiswal and Dhar, 2016; Puente-Diaz and Cavazos-Arroyo, 2017; Royston and Reiter-Palmon, 2019; Yang et al., 2020). Moreover, several meta-analyses of creativity have demonstrated that creative self-efficacy exhibited a moderately strong relationship with creativity (Hammond et al., 2011; Ng and Feldman, 2012). Based on the aforementioned studies, creative self-efficacy appears to be essential for creativity.

According to social cognitive theory, the family environment is a primary source of self-efficacy (Bandura, 1997). Empirically, Turner et al. (2009) found that authoritative parents tended to encourage their children to be autonomous and communicated effectively with their children, which contributed to higher academic efficacy. Later studies also indicated that an authoritative parenting style was positively related to self-efficacy (Masud et al., 2016; Llorcamestre et al., 2017). Importantly, there was evidence directly showing that perceived parental support was linked with heightened creative self-efficacy (Gralewski and Jankowska, 2020; Chen et al., 2021). Considering the above, it seems reasonable that parental responsiveness, characterized by attentive, warm reactions toward children’s needs to foster individuality, self-regulation, and self-assertion, can facilitate creative self-efficacy. This responsiveness may help children independently handle creative problems more effectively and feel more confident in their creative ability to overcome obstacles.

Some researchers have perceived teachers as another influential source of students’ self-efficacy (Bandura, 1997). Rosenfeld et al. (2000) investigated the effect of social support from parents, teachers, and friends on self-efficacy. The researchers’ results suggested the centrality of the teacher in social support in promoting self-efficacy. Choi (2004) further showed that the extent to which instructors supported students’ participation and ideas was positively related to students’ creative self-efficacy. In the same vein, researchers found that students’ creative self-efficacy could be enhanced by supportive teacher feedback (Beghetto, 2006), supportive supervisory style (Gu et al., 2017), and teacher support (Sun et al., 2021). These findings imply that teaching responsiveness may benefit the creative self-efficacy of students.

In conclusion, previous studies have provided evidence for a positive relationship between parental responsiveness (Miller et al., 2012; Mehrinejad et al., 2015; Guo et al., 2021; Liang et al., 2021), teaching responsiveness (Amabile, 1989; Davies et al., 2013; Richardson and Mishra, 2018; Du et al., 2019; Gao et al., 2020), and creativity. Moreover, creative self-efficacy may mediate these associations and explain how environmental responsiveness correlates with creativity. The present study was designed to investigate the relationships between parental responsiveness, teaching responsiveness, and creativity, along with the underlying mechanism. Building on theoretical links and empirical studies, we made the following hypotheses:

*Hypothesis 1*: Parental responsiveness (1a) and teaching responsiveness (1b) are positively related to creativity.

*Hypothesis 2*: Creative self-efficacy mediates the relationships between parental responsiveness, teaching responsiveness, and creativity.

## Materials and Methods

### Participants

We adopted a convenience sampling method and administered self-report questionnaire surveys to the participants. A total of 606 college students participated in this study, 22 of whom were excluded because of errors such as numerous missing values and outliers. The final sample consisted of 584 college students (419 women and 165 men) at two universities in Chengdu (*N* = 304; 52%) and Beijing (*N* = 280; 48%) in China. The participants’ ages ranged from 17 to 24 years (*M* = 19.11 years, *SD* = 1.21 years).

Before beginning data collection, we explained the purpose of the research to the students and obtained their consent. Next, we gave the students a multi-section questionnaire, which they completed in a quiet classroom environment. The study was anonymous to protect the participants’ private information.

### Measures

Because the original version of the questionnaire was written in English, two primary translation steps were adopted to guarantee the validity and accuracy of the translated version. First, two professional researchers in the creativity domain independently translated the original English version of the questionnaire into a Chinese version, generating two versions of the questionnaire. The translators then discussed each item and resolved any discrepancies between the two initial translated versions to generate a single consensus Chinese version. In the second step, a bilingual person who was blind to the original English version translated the items back to English. The previous two researchers compared each back-translated item with the original English version to further identify and amend any inadequate expression in the translation. We also pretested the questionnaire on the target population by inviting several students to complete the questionnaire and point out any words they did not understand. Then we adapted the identified words to conform better to the students’ usual language. The final Chinese version had the same items and structures as the English version.

In the present study, we retested the validity and reliability of the Chinese version of the questionnaire (see details in Supplementary Material). Cronbach’s alpha was used to test the reliability of the scales. The validity of the scales was measured using confirmatory factor analysis (CFA). The items that did not fulfill the criteria were deleted.

#### Creativity

The participants’ creativity was measured by the Biographical Inventory of Creative Behaviors, developed by Batey (2007). This inventory contains 34 items, covering the common domains of everyday creativity (e.g., arts, crafts, and creative writing) and social creativity (e.g., leadership, coaching, and mentorship); thus, it represents the most common kinds of everyday creative activities. The students responded to each item with a yes/no, forced-choice answer, indicating any activities they had been actively involved in during the past 12 months. The creativity score was obtained by summing the scores of all the items. This scale does not have subscales. In the present study, Cronbach’s alpha was 0.82.

#### Creative Self-Efficacy

The participants’ creative self-efficacy was measured via a creative confidence questionnaire developed by Phelan and Young (2003). The original questionnaire contains 12 items forming one factor with a 6-point Likert scale ranging from “1” (strongly disagree) to “6” (strongly agree). A sample item is “I feel confident in my ability to solve problems.” The final questionnaire consisted of seven items, and Cronbach’s alpha for the scale was 0.88.

#### Parental Responsiveness

The present study selected the parental responsiveness subscale from the Parenting Style Inventory II (Darling and Toyokawa, 1997). We translated this English version subscale into the Chinese version scale, which was also used in a previous study (Zhang et al., 2021). The original subscale consists of five items (e.g., “My most influential parent and I do things that are fun together”). The response categories range from “1” (strongly disagree) to “6” (strongly agree). In the present study’s parental responsiveness scale, the participants first indicated their most influential parent (e.g., father, mother, grandfather, and others) when they were between the ages of 15 and 19. They then completed the scale to measure their perceived parental responsiveness. The final parental responsiveness scale consisted of three items. Cronbach’s alpha for the scale was 0.65, the acceptable values of 0.7 or 0.6 due to the small number of items (Griethuijsen et al., 2015).

#### Teaching Responsiveness

For the present study, we selected the Lecture Responsiveness Scale adapted by Zhang et al. (2021) to measure teaching responsiveness. The original scale consists of five items and uses a 6-point Likert scale. A sample item is “My teachers truly care about me.” The final scale consisted of four items, and Cronbach’s alpha for the scale was 0.78.

### Data Analysis

We used structural equation modeling (SEM) to examine the mediating effect. First, we tested the measurement model to evaluate whether each latent variable predicted its indicators well. Second, a structural model was tested to estimate the latent relationships of parental responsiveness, teaching responsiveness, creative self-efficacy, and creativity. In addition, the indirect effect of environmental responsiveness on creativity via creative self-efficacy was evaluated by the bootstrapping method. All these analyses proceeded in the Mplus (version 7.1) program.

The following five fit indices were selected to evaluate the goodness of fit of the models: the chi-square divided by the degrees of freedom (χ*^2^/df*), the comparative fit index (CFI), the Tucker–Lewis index (TLI), the root mean square error of approximation (RMSEA), and the standardized root mean square residual (SRMR). The following criteria were used to assess the model fit, as suggested by Hu and Bentler (1999): values of CFI and TLI greater than 0.95, RMSEA close to 0.06 (or lower), and SRMR close to 0.08 (or lower). In addition, the value of χ*^2^/df* should be less than 3.0 (Kline, 2005).

## Results

### Descriptive Statistics and Correlations Among All the Variables

Table 1 displays the descriptive statistics and correlations among all the variables in the present study.

**TABLE 1:** Means, standard deviations, and correlations among all variables.

Variable	Mean	*SD*	1	2	3	4
1. Creativity	8.40	4.92	–			
2. CSE	4.14	0.82	0.28***	–		
3. PResp	4.26	1.12	0.15***	0.22***	–	
4. TResp	3.71	1.08	0.12**	0.23***	0.25***	–

CSE, creative self-efficacy; PResp, parental responsiveness; TResp, teaching responsiveness. **p < 0.01; ***p < 0.001.

Creativity was significantly correlated with parental responsiveness (*r* = 0.15, *p* < 0.001) and teaching responsiveness (*r* = 0.12, *p* < 0.01). Creative self-efficacy was significantly correlated with creativity (*r* = 0.28, *p* < 0.001), parental responsiveness (*r* = 0.22, *p* < 0.001), and teaching responsiveness (*r* = 0.23, *p* < 0.001). In addition, parental responsiveness was significantly correlated with teaching responsiveness (*r* = 0.25, *p* < 0.001).

### Model Specification

The correlation analysis revealed that creativity was significantly correlated with environmental responsiveness and creative self-efficacy. In accordance with the theoretical and data analysis, we established a structural model to analyze whether environmental responsiveness indirectly affected creativity through creative self-efficacy.

The measurement model consisted of three latent variables (i.e., parental responsiveness, teaching responsiveness, and creative self-efficacy). As can be seen in Table 2, it had a satisfactory fit. All the factor loadings in the model were significant (*p* < 0.001), suggesting that each latent variable was well represented by its indicators.

**TABLE 2:** Fit indices of the measurement model and structural model.

Model	χ*^2^/df*	CFI	TLI	RMSEA	SRMR
Measurement model	2.40	0.96	0.95	0.05	0.06
Structural model	1.89	0.97	0.97	0.04	0.03

*CFI, comparative fit index; TLI, Tucker–Lewis index; RMSEA, root mean square error of approximation; SRMR, standardized root mean square residual.*

**FIGURE 1:**
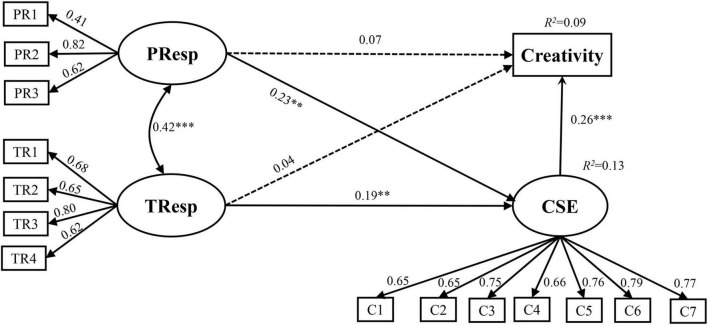
Results of SEM evaluating the mediating role of creative self-efficacy in the relationships of parental responsiveness, teaching responsiveness, and creativity. CSE, creative self-efficacy; PResp, parental responsiveness; TRsep, teaching responsiveness; PRI-3, parental responsiveness items 1–3. TR1-4, teaching responsiveness items 1–4; C1-7, creative self-efficacy items 1–7. **p < 0.01; ***p < 0.001.

Structural equation modeling was performed to estimate the latent relationships of parental responsiveness, teaching responsiveness, creative self-efficacy, and creativity. As Table 2 reveals, the structural model revealed a satisfactory fit. Figure 1 shows the SEM results, which indicated that parental responsiveness and teaching responsiveness were significant predictors of creative self-efficacy (β = 0.23, *p* < 0.001; β = 0.19, *p* < 0.01, respectively). Creative self-efficacy was a significant predictor of creativity (β = 0.26, *p* < 0.001). As for interrelationship, parental responsiveness and teaching responsiveness had significant positive correlation (*r* = 0.42, *p* < 0.001).

### The Mediating Role of Creative Self-Efficacy

We used the bootstrapping method to test the mediating role of creative self-efficacy. This procedure randomly samples with replacement to construct multiple samples from the original data set, and obtains the estimates of indirect effect and their confidence interval (CI) for the resulting sampling distribution. We generated 1,000 bootstrap samples from the original sample (*N* = 584) to test the mediating role of creative self-efficacy. Following Preacher and Hayes (2008), if the 95% CI of indirect effect does not include 0, the mediating effect is significant.

Table 3 displays the indirect effects and their related 95% CIs. According to the results, parental responsiveness and teaching responsiveness both exerted significant indirect effects on creativity via creative self-efficacy.

**TABLE 3:** Standardized indirect effects and 95% confidence intervals.

Mediation pathway	Estimate	*SE*	95% CI		*p*
			Lower	Upper	
PResp → CSE → Creativity	0.06	0.02	0.02	0.10	0.008
TResp → CSE → Creativity	0.05	0.02	0.01	0.09	0.002

*CSE, creative self-efficacy; PResp, parental responsiveness; TResp, teaching responsiveness.*

## Discussion

The present study investigated relationships between parental responsiveness, teaching responsiveness, and creativity in college students, along with the potential mechanism underlying these associations. The results demonstrated that parental responsiveness and teaching responsiveness were related to student creativity. In addition, creative self-efficacy served as a mediating variable in the relationship between environmental responsiveness and creativity.

### The Relationships of Parental Responsiveness, Teaching Responsiveness, and Creativity

In accordance with our hypothesis, parental responsiveness and teaching responsiveness were positively correlated with student creativity. That is, warmly supportive parents and teachers who offer students strong support and autonomy to explore new area of interest may improve student creativity. This outcome was reported in previous studies. For instance, various researchers have found that a parenting style with a high level of responsiveness was correlated with a high level of creativity (Miller et al., 2012; Mehrinejad et al., 2015). Other studies have suggested that a positive teacher–student relationship with warm, caring characteristics was positively associated with creativity (Davies et al., 2013; Richardson and Mishra, 2018).

The present study’s findings also revealed that parental responsiveness and teaching responsiveness were correlated with each other, which is consistent with Yeh (2004)’s assumption that the family and school experience systems interact with each other. This phenomenon may be due to two reasons. First, parents with a high level of responsiveness tend to choose teachers who exhibit supportive behavior in communicating with students. Second, students growing up in a warm, responsive family climate are more likely to seek help and guidance from their teachers, which may, in turn, increase their perceived teaching responsiveness.

### The Mediating Role of Creative Self-Efficacy in the Structural Relationships of Parental Responsiveness, Teaching Responsiveness, and Creativity

The present study further revealed the mediating role of creative self-efficacy in the relationships of parental responsiveness, teaching responsiveness, and creativity. This outcome is consistent with the ecological systems model of creativity development, which states that the family subsystem and school experience subsystem influence an individual’s creativity chiefly through personal characteristics (Yeh, 2004).

The present study’s findings demonstrated a significant indirect effect of parental responsiveness on creativity via creative self-efficacy. In other words, students perceiving more responsiveness and support from parents had higher levels of creative self-efficacy, which enhanced creativity. This outcome is consistent with previous studies. On the one hand, several previous findings revealed a positive influence of parental warmth and supportive involvement on creativity (Gralewski and Jankowska, 2020; Guo et al., 2021; Liang et al., 2021). On the other hand, many studies have demonstrated that creative self-efficacy was positively correlated with creativity (Li and Wu, 2011; Jaiswal and Dhar, 2016; Puente-Diaz and Cavazos-Arroyo, 2017; Royston and Reiter-Palmon, 2019; Yang et al., 2020). This improved creative self-efficacy, in turn, leads to perseverance when engaging in creative activities, even if the activities are time-consuming and effort-intensive.

The current study also revealed a significant positive indirect effect of teaching responsiveness on creativity through creative self-efficacy. Previous studies have revealed that creative self-efficacy mediated the relationship between teacher support (Sun et al., 2021), supervisory style (Gu et al., 2017) and creativity. Teaching responsiveness, which refers to teachers who are sensitive and responsive to students’ needs and provide warm, resourceful, encouraging creative teaching environments, makes students feel more confident in their creative capabilities (Rosenfeld et al., 2000; Beghetto, 2006). Heightened creative self-efficacy is likely to help students tackle creative problems with more endurance (Li and Wu, 2011; Royston and Reiter-Palmon, 2019; Du et al., 2020). Therefore, students who perceive more responsiveness from teachers are likely to have more creative self-efficacy, which may advance creativity.

### Implications

The present study was the first to propose and examine the significance of environmental responsiveness for creativity. Our study revealed that environmental responsiveness might influence creativity via creative self-efficacy, providing further insight into the mechanism of the associations among these factors. All these results provide empirical evidence for developing creativity from the perspective of environmental factors.

From a practical viewpoint, one method of fostering student creativity is to immerse students in a social environment that improves creativity (Soh, 2017). Home and school are two primary developmental contexts (Walker and Hoover-Dempsey, 2006); thus, parents and teachers play a central role in fostering creativity. According to the present study’s findings, teachers and parents should seek to provide a warm response to the creative needs of students, which will help students establish a high level of confidence and attain creative achievement. Teachers might achieve this end by offering students opportunities to work with tasks creatively, instructing them to deal with frustration and failure while in trouble, encouraging and reinforcing their creative behavior whiling solving tough tasks. The students also need a warm response from their parents. Lim and Smith (2008) emphasized that “tolerance and encouragement from parents represents an initial and continually influential environment in which the development and maintenance of creativity is dependent upon” (p. 413). In other words, parents’ tolerance of novelty, encouragement of independence, and support of creative needs are essential for student creativity. Moreover, the significant relationship between parental responsiveness and teaching responsiveness underscores the importance of the home–school community. Policymakers could pay attention to environmental responsiveness and enhance the interaction of family and school to foster student creativity when making policies. For example, school leaders can create formal training programs focusing on environmental responsiveness for parents and teachers together. These programs will not only promote their skills in supporting students but also strengthen the cooperation between teachers and parents.

### Limitations and Future Research

Although the present study identified the significance of environmental responsiveness for creativity, a few limitations should be mentioned. First, this study was the first to investigate the relationship between environmental responsiveness and creativity; hence, more studies should be conducted to verify or compare the present results. For instance, results may vary across cultures. According to Bronfenbenner (1979), “Within any culture or subculture, settings of a given kind—such as homes, streets, or offices—tend to be very much alike, whereas between cultures they are distinctly different” (p. 4). Nonetheless, previous studies across different cultures have implied similar results. In Eastern counties, creative self-efficacy was found to mediate the relationship between perceived teacher support and students’ creativity (Sun et al., 2021). In Western countries, evidence was also reported indicating a positive relationship between supportive leadership, creative self-efficacy, and creativity (Choi, 2004). Due to the contradiction between theoretical predictions and empirical results, more studies among cultures should be conducted to obtain reliable and solid findings.

Additionally, it should be noted that the participants were college students. Any generalization of the current results to other age groups should be made with caution. Research has shown that the ecological system of the family and school system might have a great direct effect on the creativity of children and teens (Yeh, 2004). As they grow up, the effect becomes more indirect. Our findings verified that, compared to children and teens, environmental responsiveness shaped people’s creativity through personal factors such as creative self-efficacy. According to this view, the mechanisms for how environmental responsiveness shapes individuals’ creativity might conceivably differ for people in different age groups. Therefore, future research should examine the relationships between environmental responsiveness, creative self-efficacy, and creativity in cross-sectional studies with a wide range of ages, even in longitudinal studies, to obtain more nuanced results.

## Conclusion

The present study found a positive relationship between environmental responsiveness and creativity. Moreover, creative self-efficacy mediated the relationship. These findings show the significance of environmental responsiveness for creativity, implying that parents and teachers can foster student creativity by caring for and responding to students’ creative needs. According to this view, parents, teachers, and policymakers should attach importance to environmental responsiveness and promote creativity by providing warm and supportive responses to students.

## Data Availability Statement

The raw data supporting the conclusions of this article will be made available by the authors, without undue reservation.

## Ethics Statement

The studies involving human participants were reviewed and approved by Scientific Research Ethics Committee, Institute of Psychology, Chinese Academy of Sciences. The patients/participants provided their written informed consent to participate in this study.

## Author Contributions

YZ: conceptualization, methodology, formal analysis, visualization, data curation, and writing – original draft. PL: conceptualization, investigation, formal analysis, visualization, and writing – original draft. ZZ: resources, review, and editing. XZ: review, editing, and supervision. JS: review, editing, supervision, and project administration. All authors contributed to the article and approved the submitted version.

## Conflict of Interest

The authors declare that the research was conducted in the absence of any commercial or financial relationships that could be construed as a potential conflict of interest.

## Publisher’s Note

All claims expressed in this article are solely those of the authors and do not necessarily represent those of their affiliated organizations, or those of the publisher, the editors and the reviewers. Any product that may be evaluated in this article, or claim that may be made by its manufacturer, is not guaranteed or endorsed by the publisher.

## References

[B1] Amabile T. M. (1989). *Growing up Creative: Nurturing a Lifetime of Creativity.* Amherst, MA: CEF Press.

[B2] Baker J. A. Clark T. P. Crowl A. Carlson J. S. (2009). The Influence of Authoritative Teaching on Children’s School Adjustment Are Children with Behavioural Problems Differentially Affected? *Sch. Educ. Psychol.* 30 374–382. 10.1177/0143034309106945

[B3] Bandura A. (1997). *Self-Efficacy: The Exercise of Control.* New York, NY: W. H. Freeman and Company.

[B4] Batey M. D. (2007). *A Psychometric Investigation of Everyday Creativity.* Ph.D thesis, London: University of London.

[B5] Baumrind D. (1991). The Influence of Parenting Style on Adolescent Competence and Substance Use. *J. Early Adolesc.* 11 56–95. 10.1177/0272431691111004

[B6] Beghetto R. A. (2006). Creative Self-Efficacy: correlates in Middle and Secondary Students. *Creat. Res. J.* 18 447–457. 10.1207/s15326934crj1804_4

[B7] Bogenschneider K. Pallock L. (2008). Responsiveness in Parent-Adolescent Relationships: are Influences Conditional? Does the Reporter Matter? *J. Marriage Fam.* 70 1015–1029. 10.1111/j.1741-3737.2008.00543.x

[B8] Bronfenbenner U. (1979). *The Ecology of Human Development: Experiments by Nature and Desing.* Cambridge, MA: Harvard University Press.

[B9] Campbell K. Thoburn J. W. Leonard H. D. (2017). The mediating effects of stress on the relationship between mindfulness and parental responsiveness. *Couple Family Psychol.* 6 48–59. 10.1037/cfp0000075

[B10] Chen P. Zhang J. Xu N. Zhang K. Xiao L. (2021). The relationship between need for cognition and adolescents’ creative self-efficacy: the mediating roles of perceived parenting behaviors and perceived teacher support. *Curr. Psychol.* 10.1007/s12144-021-02122-7 [Epub ahead of print].

[B11] Choi J. N. (2004). Individual and Contextual Predictors of Creative Performance: the Mediating Role of Psychological Processes. *Creat. Res. J.* 16 187–199. 10.1080/10400419.2004.9651452

[B12] Darling N. Toyokawa T. (1997). *Construction and Validation of the Parenting Style Inventory II (PSI-II).* Available Online At: https://www2.oberlin.edu/faculty/ndarling/lab/psiii.pdf (accessed March 26, 1997).

[B13] Davies D. Jindalsnape D. Collier C. Digby R. Hay P. Howe A. (2013). Creative learning environments in education : a systematic literature review. *Think. Skills Creat.* 8 80–91. 10.1016/j.tsc.2012.07.004

[B14] Dever B. V. Karabenick S. A. (2011). Is authoritative teaching beneficial for all students? A multi-level model of the effects of teaching style on interest and achievement. *Sch. Psychol. Q.* 26 131–144. 10.1037/a0022985

[B15] Du K. Wang Y. Ma X. Luo Z. Wang L. Shi B. (2020). Achievement goals and creativity: the mediating role of creative self-efficacy. *Educat. Psychol.* 40 1249–1269. 10.1080/01443410.2020.1806210

[B16] Du Y. L. Xie L. Y. Zhong J. A. Zou H. Law R. Yan X. B. (2019). Creativity fostering teacher behavior on student creative achievement: mediation of intrinsic motivation and moderation of openness to experience. *Sch. Psychol. Int.* 40 525–542. 10.1177/0143034319868271

[B17] Ertesvag S. K. (2011). Measuring authoritative teaching. *Teach. Teach. Educat.* 27 51–61. 10.1016/j.tate.2010.07.002

[B18] Fearon D. D. Copeland D. Saxon T. F. (2013). The Relationship Between Parenting Styles and Creativity in a Sample of Jamaican Children. *Creat. Res. J.* 25 119–128. 10.1080/10400419.2013.752287

[B19] Gao Q. Chen P. Zhou Z. Jiang J. (2020). The impact of school climate on trait creativity in primary school students: the mediating role of achievement motivation and proactive personality. *Asia Pacific J. Educat.* 40 330–343. 10.1080/02188791.2019.1707644

[B20] Gralewski J. Jankowska D. M. (2020). Do parenting styles matter? Perceived dimensions of parenting styles, creative abilities and creative self-beliefs in adolescents. *Think. Skills Creat.* 38:100709. 10.1016/j.tsc.2020.100709

[B21] Griethuijsen R. A. L. F. Eijck M. W. Haste H. den Brok P. J. Skinner N. C. Mansour N. (2015). Global Patterns in Students’ Views of Science and Interest in Science. *Res. Sci. Educat.* 45 581–603. 10.1007/s11165-014-9438-6

[B22] Gu J. He C. Liu H. (2017). Supervisory styles and graduate student creativity: the mediating roles of creative self-efficacy and intrinsic motivation. *Stud. High. Educat.* 42 721–742. 10.1080/03075079.2015.1072149

[B23] Guo J. J. Zhang J. Pang W. G. (2021). Parental warmth, rejection, and creativity: the mediating roles of openness and dark personality traits. *Personal. Individ. Differ.* 168:110369. 10.1016/j.paid.2020.110369

[B24] Hammond M. M. Neff N. L. Farr J. L. Schwall A. R. Zhao X. (2011). Predictors of Individual-Level Innovation at Work: a Meta-Analysis. *Psychol. Aesthet. Creat. Arts* 5 90–105. 10.1037/a0018556

[B25] Hennessey B. A. Amabile T. M. (2010). Creativity. *Annu. Rev. Psychol.* 61 569–598. 10.1146/annurev.psych.093008.100416 19575609

[B26] Hu L.t Bentler P. M. (1999). Cutoff criteria for fit indexes in covariance structure analysis: conventional criteria versus new alternatives. *Struct. Equ. Model.* 6 1–55. 10.1080/10705519909540118

[B27] Jaiswal N. K. Dhar R. L. (2016). Fostering Employee Creativity through Transformational Leadership: moderating Role of Creative Self-Efficacy. *Creat. Res. J.* 28 367–371. 10.1080/10400419.2016.1195631

[B28] Kaufman J. C. Beghetto R. A. (2009). Beyond big and little: the four c model of creativity. *Rev.Gener. Psychol.* 13 1–12. 10.1037/a0013688

[B29] Kline R. B. (2005). *Principles and Practice of Structural Equation Modeling.* New York, NY: The Guilford Press.

[B30] Li C.-H. Wu J.-J. (2011). The structural relationships between optimism and innovative behavior: understanding potential antecedents and mediating effects. *Creat. Res. J.* 23 119–128. 10.1080/10400419.2011.571184

[B31] Liang Q. L. Niu W. H. Cheng L. Qin K. X. (2021). Creativity Outside School: the Influence of Family Background, Perceived Parenting, and After-school Activity on Creativity. *J. Creat. Behav.* 205 56–60.

[B32] Lim S. Smith J. S. (2008). The Structural Relationships of Parenting Style, Creative Personality, and Loneliness. *Creat Res. J.* 20 412–421. 10.1080/10400410802391868

[B33] Liu D. Jiang K. Shalley C. E. Keem S. Zhou J. (2016). Motivational mechanisms of employee creativity: a meta-analytic examination and theoretical extension of the creativity literature. *Org. Behav. Hum. Decis. Process.* 137 236–263. 10.1016/j.obhdp.2016.08.001

[B34] Llorcamestre A. Richaud M. C. Malondavidal E. (2017). Parenting, Peer Relationships, Academic Self-efficacy, and Academic Achievement: direct and Mediating Effects. *Front. Psychol.* 8:2120. 10.3389/fpsyg.2017.02120 29326615 PMC5736920

[B35] Maccoby E. E. Martin J. A. (1983). “Socialization in the context of the family: Parent-child interaction,” in *Handbook of Child Psychology*, eds Mussen P. H. Hetherington E. M. (New York, NY: Wiley), 1–101.

[B36] Masud H. Ahmad M. S. Jan F. A. Jamil A. (2016). Relationship between parenting styles and academic performance of adolescents: mediating role of self-efficacy. *Asia Pacific Educat. Rev.* 17 121–131. 10.1007/s12564-015-9413-6

[B37] Mehrinejad S. A. Rajabimoghadam S. Tarsafi M. (2015). The Relationship between Parenting Styles and Creativity and the Predictability of Creativity by Parenting Styles. *Proc. Soc. Behav. Sci. U.S.A.* 205 56–60. 10.1016/j.sbspro.2015.09.014

[B38] Miller A. L. Lambert A. D. Neumeister K. L. S. (2012). Parenting Style, Perfectionism, and Creativity in High-Ability and High-Achieving Young Adults. *J. Educat.Gifted* 35 344–365. 10.1177/0162353212459257

[B39] Ng T. W. Feldman D. C. (2012). A comparison of self-ratings and non-self-report measures of employee creativity. *Hum. Relat.* 65 1021–1047. 10.1177/0018726712446015

[B40] Phelan S. Young A. M. (2003). Understanding creativity in the workplace: an examination of individual styles and training in relation to creative confidence and creative self-leadership. *J. Creat. Behav.* 37 266–281. 10.1002/j.2162-6057.2003.tb00994.x

[B41] Preacher K. J. Hayes A. F. (2008). Asymptotic and resampling strategies for assessing and comparing indirect effects in multiple mediator models. *Behav. Res. Methods* 40 879–891. 10.3758/brm.40.3.879 18697684

[B42] Puente-Diaz R. Cavazos-Arroyo J. (2017). Creative Self-Efficacy: the Influence of Affective States and Social Persuasion as Antecedents and Imagination and Divergent Thinking as Consequences. *Creat. Res. J.* 29 304–312. 10.1080/10400419.2017.1360067

[B43] Richards R. (2010). “Everyday creativity: Process and way of life-Four key issues,” in *Cambridge Handbook of Creativity*, eds Kaufman J. C. Sternberg R. J. (New York, NY: Cambridge University Press), 189–215.

[B44] Richardson C. Mishra P. (2018). Learning environments that support student creativity: developing the SCALE. *Think. Skills Creat.* 27 45–54. 10.1016/j.tsc.2017.11.004

[B45] Rosenfeld L. B. Richman J. M. Bowen G. L. (2000). Social Support Networks and School Outcomes: the Centrality of the Teacher. *Tradition* 17 205–226. 10.1023/A:1007535930286

[B46] Royston R. Reiter-Palmon R. (2019). Creative self-efficacy as mediator between creative mindsets and creative problem-solving. *J. Creat. Behav.* 53 472–481. 10.1002/jocb.226

[B47] Sawyer K. R. (2012). *Explaining Creativity: The Science of Human Innovation.* New York, NY: Oxford University Press, Inc.

[B48] Soh K. (2017). Fostering student creativity through teacher behaviors. *Think. Skills Creat.* 23 58–66. 10.1016/j.tsc.2016.11.002

[B49] Sun C. C. Zhou Z. J. Yu Q. L. Gong S. Y. Yi L. Cao Y. (2021). Exploring the Effect of Perceived Teacher Support on Multiple Creativity Tasks: based on the Expectancy-Value Model of Achievement Motivation. *J. Creat. Behav.* 55 15–24. 10.1002/jocb.430

[B50] Tierney P. Farmer S. M. (2002). Creative Self-Efficacy: its Potential Antecedents and Relationship to Creative Performance. *Acad. Manage. J.* 45 1137–1148. 10.5465/3069429 3069429

[B51] Torrance E. P. (1966). Nurture of creative talents. *Theor. Pract.* 5 167–173. 10.1080/00405846609542020

[B52] Turner E. A. Chandler M. Heffer R. W. (2009). The Influence of Parenting Styles, Achievement Motivation, and Self-Efficacy on Academic Performance in College Students. *J. College Stud. Devel.* 50 337–346. 10.1353/csd.0.0073 34409987

[B53] Walker J. M. (2008). Looking at teacher practices through the lens of parenting style. *J. Exp. Educat.* 76 218–240. 10.3200/JEXE.76.2.218-240

[B54] Walker J. M. T. Hoover-Dempsey K. V. (2006). “Why research on parental involvement is important to classroom management,” in *The Handbook of Classroom Management*, eds Evertson C. M. Weinstein C. S. (New York, NY: Routledge), 665–684.

[B55] Yang Y. Xu X. Liu W. Pang W. (2020). Hope and Creative Self-Efficacy as Sequential Mediators in the Relationship Between Family Socioeconomic Status and Creativity. *Front. Psychol.* 11:438. 10.3389/fpsyg.2020.00438 32256427 PMC7090163

[B56] Yeh Y.-C. (2004). The Interactive Influences of Three Ecological Systems on R & D Employees’. *Technol. Creat. Creat. Res. J.* 16 11–25. 10.1207/s15326934crj1601_2

[B57] Zhang Z. S. Hoxha L. Aljughaiman A. Arenliu A. Gomez-Arizaga M. P. Gucyeter S. (2021). Social Environmental Factors and Personal Motivational Factors Associated with Creative Achievement: a Cross-Cultural Perspective. *J. Creat. Behav.* 55 410–432. 10.1002/jocb.463

