# Supplementary material for: The Relationships of Parental Responsiveness, Teaching Responsiveness, and Creativity: The Mediating Role of Creative Self-Efficacy
Source: Front Psychol. 2022 Feb 4;12:748321. doi: 10.3389/fpsyg.2021.748321 (PMC8855146; doi:10.3389/fpsyg.2021.748321)
Supplement: Supplementary file 1 [file Table_1.docx]

**Supplementary Material**

**Section 1 The Validation Process of the Chinese Version of the Questionnaire**

In the present study, we retested the validity and reliability of the Chinese version of the questionnaire. Cronbach’s alpha was used to test the reliability of the scales. The validity of the scales was measured using confirmatory factor analysis (CFA). Three criteria had to be met to obtain a satisfactory CFA model:

1. The factor loading was higher than 0.5 (and the lowest criterion was 0.4).

2. The goodness-of-fit indices met the recommended thresholds.

3. The number of items for one factor was at least 3.

The following five fit indices were selected to evaluate the goodness of fit of the CFA models: the Chi-square divided by the degrees of freedom (*χ^2^/df*), the comparative fit index (CFI), the Tucker–Lewis index (TLI), the root mean square error of approximation (RMSEA), and the standardized root-mean-square residual (SRMR). The following criteria were used to assess the model fit (Hu & Bentler, 1999): the values of CFI and TLI should be greater than 0.95, RMSEA was close to 0.06 (or lower), and SRMR was close to 0.08 (or lower). In addition, the value of *χ2/df* should be less than 3.0 (Kline, 2005).

The items that did not fulfill these criteria were deleted. We deleted five items from the creative self-efficacy scale, two items from the parental responsiveness scale, and one item from the teaching responsiveness scale.

**The validity and reliability of the Chinese version of the questionnaire**

Following the above criteria, the final model of creative self-efficacy, parental responsiveness, and teaching responsiveness was analyzed and presented in this section.

**Table 1**

*Fit Indices and Reliability of the Creative Self-Efficacy Model, Parental Responsiveness Model, Teaching Responsiveness Model*

| Scale | *χ2/df* | CFI | TLI | RMSEA | SRMR | Cronbach’s alpha |
| --- | --- | --- | --- | --- | --- | --- |
| Creative Self-Efficacy | 2.98 | 0.98 | 0.98 | 0.06 | 0.02 | 0.88 |
| Parental Responsiveness | -- | 1.00 | 1.00 | 0.00 | 0.00 | 0.65 |
| Teaching Responsiveness | 2.12 | 1.00 | 0.99 | 0.04 | 0.01 | 0.78 |

*Note*. *N* = 584.

**Table 2**

*Factor Loadings for Items on the* *Creative Self-Efficacy Scale*

| Item | Factor loading |
| --- | --- |
| I feel confident in my ability to solve problems. | **0.65** |
| I feel confident in my ability to choose the best alternative. | **0.65** |
| I feel confident in my ability to get my ideas implemented. | **0.75** |
| I feel confident in my ability to create desired changes effectively. | **0.66** |
| I feel confident in my ability to improve products or services. | **0.76** |
| When I take on a project, I am certain I can create great outcomes. | **0.79** |
| I feel confident in my ability to invent new products or processes. | **0.73** |

*Note*. Factor loadings *>* 0.50 are bold.

For the final creative self-efficacy model, summaries of the model fit indices, factor loadings, and Cronbach’s alpha are presented in Table 1 and Table 2. The creative self-efficacy model showed an acceptable fit. Cronbach’s alpha coefficient was 0.88, which was acceptable.

**Table 3**

*Factor Loadings for Items on the Parental Responsiveness Scale*

| Item | Factor loading |
| --- | --- |
| My most influential parent and I do things together that are fun. | **0.71** |
| My most influential parent spends time just talking to me. | **0.71** |
| My most influential parent hardly ever praises me for doing well. | 0.47 |

*Note*. Factor loadings *>* 0.50 are bold.

Cronbach’s alpha and factor loadings are presented in Table 1 and Table 3 for the final parental responsiveness model. A minimum of three indicators is required for one-factor models. When three indicators are used, the one-factor model is justified, and goodness-of-fit indices do not apply (Brown, 2015). Thus, the final model retained one item whose factor loading was less than 0.50, but it was higher than 0.40, which was acceptable (Wu, 2010). The Cronbach’s alpha coefficient was 0.65.

**Table 4**

*Factor Loadings for Items on the Teaching Responsiveness Scale*

| Item | Factor loading |
| --- | --- |
| My lecturers talk to me about my daily life beyond class time. | **0.70** |
| My lecturers join me to participate in extracurricular activities, such as playing basketball. | **0.63** |
| My lecturers truly care about me. | **0.78** |
| My lecturers comfort me when I do not perform well. | **0.65** |

*Note*. Factor loadings *>* 0.50 are bold.

The model fit indices, factor loadings, and Cronbach’s alpha are presented in Table 1 and Table 4 for the final teaching responsiveness model. The teaching responsiveness model showed an acceptable fit. Cronbach’s alpha coefficient was 0.78, which was an acceptable internal consistency for the scale.

**Section 2 The Example of Validation Process: The Parental Responseness Scale**

In this section, we tested the validity and reliability of the parental responsiveness scale as an example of the validation process for measurement instruments.

Concerning the parental responsiveness scale, it was a subscale from the Parenting Style Inventory II (Darling and Toyokawa, 1997). We translated this English version subscale into the Chinese version scale, which was also used in a previous study (Zhang et al., 2021). In the present study, we retested this scale and deleted two inappropriate items. We compared the validity and reliability between the original and final parental responsiveness scale to explain why removed these two items.

**Table 5**

*Fit Indices and Reliability of the Original Model and Final Model*

| Scale | *χ2/df* | CFI | TLI | RMSEA | SRMR | Cronbach’s alpha |
| --- | --- | --- | --- | --- | --- | --- |
| Original Model | 10.502 | 0.881 | 0.763 | 0.128 | 0.053 | 0.64 |
| Final Model | -- | 1.00 | 1.00 | 0.00 | 0.00 | 0.65 |

*Note*. *N* = 584.

**Table 6**

*Factor Loadings for Items on the Original and Final Parental Responsiveness Scale*

| Item | Original scale | Final scale |
| --- | --- | --- |
| My most influential parent and I do things together that are fun. | **0.66** | **0.71** |
| My most influential parent spends time just talking to me. | **0.74** | **0.71** |
| My most influential parent hardly ever praises me for doing well. | 0.48 | 0.47 |
| I can count on my most influential parent to help me out if I have a problem. | 0.41 | -- |
| My most influential parent doesn’t really like me to tell them my troubles. | 0.26 | -- |

*Note*. Factor loadings *>* 0.50 are bold.

Cronbach’s alpha and factor loadings are presented in Table 5 and Table 6 for the original and final parental responsiveness model.

For the original model, the model fit was not satisfactory, and there are three items whose factor loadings were less than 0.50. A minimum of three indicators is required for a one-factor model. Thus, the final model retained one item whose factor loading was less than 0.50, but it was higher than 0.40, which was acceptable (Wu, 2010). The Cronbach’s alpha coefficient was 0.65, the acceptable values of 0.7 or 0.6 due to the small number of items (Griethuijsen et al., 2015).

**References**

Brown, T. A. (2015). *Confirmatory factor analysis for applied research*. (2th ed.). New York: The Guildford Press.

Darling, N., and Toyokawa, T. (1997). *Construction and validation of the parenting style inventory II (PSI-II)* [Online]. Available: https://www2.oberlin.edu/faculty/ndarling/lab/psiii.pdf [Accessed 3/26/1997].

Griethuijsen, R.A.L.F., Eijck, M.W., Haste, H., den Brok, P.J., Skinner, N.C., Mansour, N., et al. (2015). Global Patterns in Students’ Views of Science and Interest in Science. *Research in Science Education* 45(4), 581-603. doi: 10.1007/s11165-014-9438-6.

Hu, L.t., and Bentler, P.M. (1999). Cutoff criteria for fit indexes in covariance structure analysis: Conventional criteria versus new alternatives. *Structural equation modeling: a multidisciplinary journal* 6(1), 1-55. doi: 10.1080/10705519909540118.

Kline, R.B. (2005). *Principles and practice of structural equation modeling*. New York: The Guilford Press.

Wu, M.-l. (2010). *Questionnaire statistical analysis practice--SPSS operation and application*. Chongqing: Chongqing University Press.

Zhang, Z.S., Hoxha, L., Aljughaiman, A., Arenliu, A., Gomez-Arizaga, M.P., Gucyeter, S., et al. (2021). Social Environmental Factors and Personal Motivational Factors Associated with Creative Achievement: A Cross-Cultural Perspective. *Journal of Creative Behavior* 55(2), 410-432. doi: 10.1002/jocb.463.
